# Genetic diversity across the mitochondrial genome of eastern oysters (*Crassostrea virginica*) in the northern Gulf of Mexico

**DOI:** 10.7717/peerj.12205

**Published:** 2021-09-28

**Authors:** Chani R. Rue, Jason D. Selwyn, Patricia M. Cockett, Bryan Gillis, Lauren Gurski, Philip Jose, Brandi L. Kutil, Sharon F. Magnuson, Luz Ángela López de Mesa, R Deborah Overath, Delbert Lee Smee, Christopher E. Bird

**Affiliations:** 1 Department of Life Sciences, Texas A&M University—Corpus Christi, Corpus Christi, TX, United States of America; 2 Harte Research Institute, Texas A&M University—Corpus Christi, Corpus Christi, TX, United States of America; 3 Conrad Blucher Institute, Texas A&M University—Corpus Christi, Corpus Christi, TX, United States of America; 4 Department of Undergraduate Studies, Texas A&M University—Corpus Christi, Corpus Christi, TX, United States of America; 5 Department of Mathematics and Sciences, Texas Southmost College, Brownsville, TX, United States of America; 6 Dauphin Island Sea Lab, Dauphin Island, AL, United States of America; 7 Marine Sciences, University of South Alabama, Mobile, AL, United States of America; 8 Hawai‘i Institute of Marine Biology, University of Hawaii at Mānoa, Kāne‘ohe, Hawai‘i, United States of America

**Keywords:** ezRAD, Mitochondrial genome, *Crassostrea virginica*, Sanger sequencing

## Abstract

The eastern oyster, *Crassostrea virginica*, is divided into four populations along the western North Atlantic, however, the only published mitochondrial genome sequence was assembled using one individual in Delaware. This study aimed to (1) assemble *C. virginica* mitochondrial genomes from Texas with pooled restriction-site-associated DNA sequencing (ezRAD), (2) evaluate the validity of the mitochondrial genome assemblies including comparison with Sanger sequencing data, and (3) evaluate genetic differentiation both between the Delaware and Texas genomes, as well as among three bays in Texas. The pooled-genome-assembled-genomes (PAGs) from Texas exhibited several characteristics indicating that they were valid, including elevated nucleotide diversity in non-coding and the third position of codons, placement as the sister haplotype of the genome from Delaware in a phylogenetic reconstruction of *Crassostrea* mitochondrial genomes, and a lack of genetic structure in the ND4 gene among the three Texas bays as was found with Sanger amplicons in samples from the same bays several years prior. In the comparison between the Delaware and Texas genome, 27 of 38 coding regions exhibited variability between the two populations, which were differentiated by 273 mutations, versus 1–13 mutations among the Texas samples. Using the full PAGs, there was no additional evidence for population structure among the three Texas bays. While population genetics is rapidly moving towards larger high-density datasets, studies of mitochondrial DNA (and genomes) can be particularly useful for comparing historic data prior to the modern era of genomics. As such, being able to reliably compile mitochondrial genomes from genomic data can improve the ability to compare results across studies.

## Introduction

Eastern oysters are an ecologically important foundation species that creates habitat and promotes biodiversity of fish and invertebrates (NOAA, 2007; Kroeger, 2012). Oyster reefs provide many benefits to the ecosystem and human economics, but over-extraction and habitat degradation have led to the worldwide collapse of many reefs (Beck et al., 2011; Grabowski et al., 2012). The importance of oyster reefs has focused research efforts on the effects of their loss on marine communities and ways to restore oyster reefs (Gregalis, Johnson & Powers, 2009; Rezek et al., 2017). Oyster reefs diversify the landscape and stabilize the shoreline, reducing the impacts from erosion and flooding (Grabowski et al., 2012). Further, oysters improve water quality and reduce turbidity through denitrification, removal of chlorophyll and bacterial biomass, and by recycling nutrients (NOAA, 2007; Kroeger, 2012). Yet, with the presence of global oyster industries, only 15% of the world’s natural oyster reef habitat remains (Kroeger, 2012).

In addition to being ecologically important, oysters have been an economically and nutritionally important fishery throughout human history in the Gulf of Mexico (Ricklis, 1996). Oysters were first locally overexploited in North America in the early 1600′s and various management plans and laws have been passed since then including some of the earliest in 1658 and 1679 (Kirby, 2004). Over time, the center of the fishery for *C. virginica* has shifted from the mid-Atlantic region to the Gulf of Mexico as stocks are depleted (Kirby, 2004; Tunnell, 2017). In the Gulf of Mexico, oysters rank as the second most valuable shellfish fishery (Tunnell, 2017). In 2017, the Gulf of Mexico yielded more than 8,000 metric tons of oysters, over 73% of the total U.S. production (NOAA Fisheries Office of Science and Technology, 2019). Within this region, Texas supplied about 20% to Gulf coast production and about 14% to overall US production (NOAA Fisheries Office of Science and Technology, 2019). To maintain this resource, it is crucial to develop conservation strategies that promote sustainable harvest and the maintenance of genetic diversity in the face of human-induced population bottlenecks. In order to develop successful management plans, understanding the biology and population connectivity patterns of *C. virginica* in the Gulf of Mexico is important (Beck et al., 2011).

**Figure 1:**
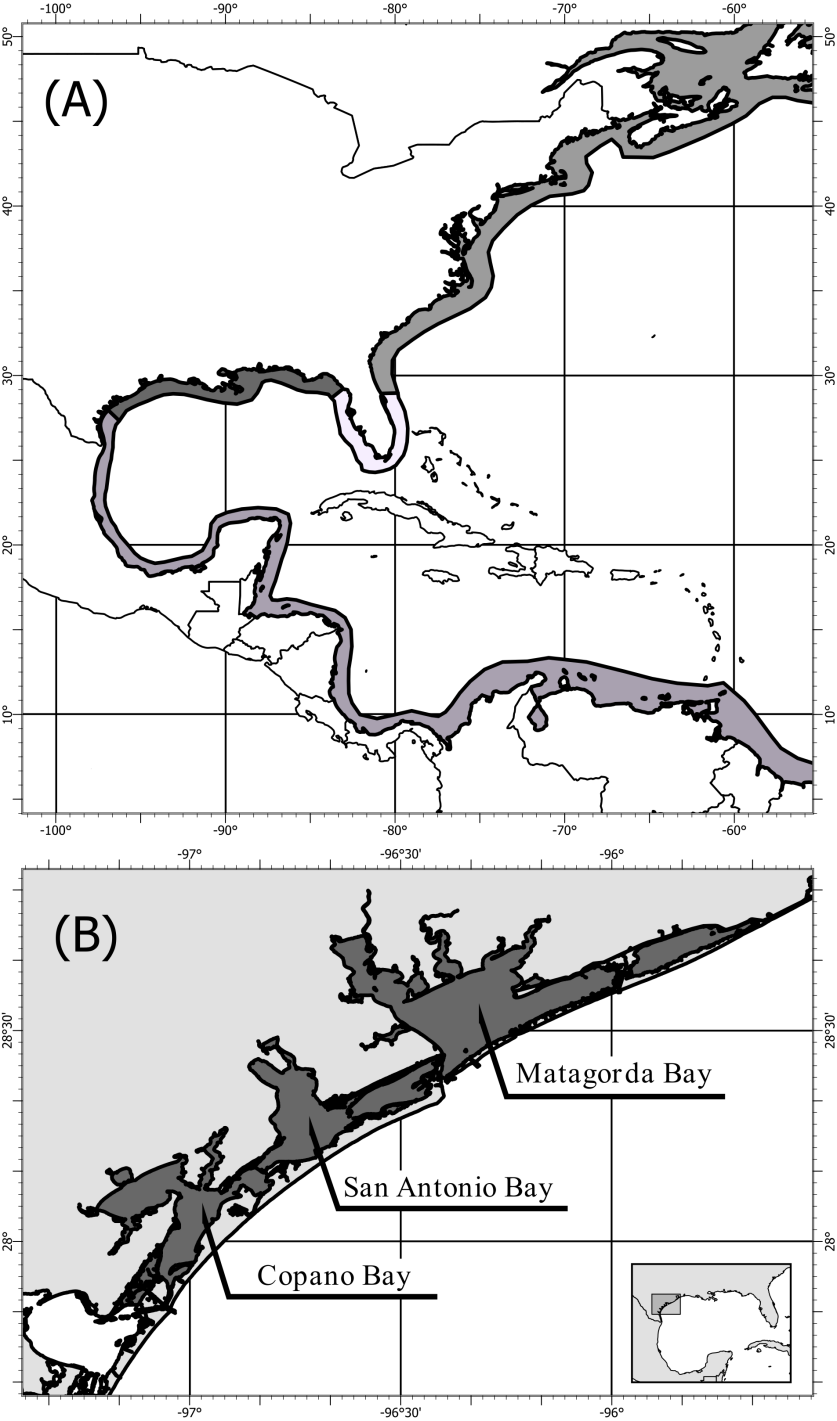
Map of population structure of *C. virginica* and sampling locations. Map (A) showing the distribution of *C. virginica* including areas of observed genetic breaks separating the four distinct populations. Map (B) of the sampling locations of this study along the Coastal Bend of Texas, USA, including: Copano, Matagorda, and San Antonio Bays, near the boundary between the Western Gulf of Mexico and Central/South American populations.

*Crassostrea virginica* (Gmelin, 1791) populations span the length of the North American coast from St. Lawrence Bay, Canada to the Yucatan Peninsula, Mexico (Galtsoff, 1964; Fig. 1A). Four genetically distinct populations have been identified using mitochondrial (Reeb & Avise, 1990; Hare & Avise, 1996; Varney et al., 2009) and nuclear DNA (Buroker, 1983; Karl & Avise, 1992; King, Ward & Zimmerman, 1994; Hare & Avise, 1996; Hoover & Gaffney, 2005; Anderson et al., 2014), showing three population boundaries: (1) the Atlantic, (2) the Eastern Gulf of Mexico, and (3) the Western Gulf of Mexico (Fig. 1A). The population boundary in the Western Gulf of Mexico, located near Corpus Christi and Port Aransas, Texas, is shifting northward as the Central/South American population expands into the Gulf of Mexico (King, Ward & Zimmerman, 1994; Varney et al., 2009; Anderson et al., 2014). Despite multiple distinct population segments only one complete mitochondrial DNA genome of the eastern oyster (*Crassostrea virginica*) is available in the U.S. National Center for Biotechnology Information, GenBank (Milbury & Gaffney, 2005). This oyster was collected in Delaware along the Atlantic coast, and the mitochondrial genome was sequenced as part of assembling the entire *C. virginica* genome (Gómez-Chiarri et al., 2015).

Next-generation sequencing (NGS) is becoming increasingly wide-spread in population genetic studies; however, historic research has often been performed using single or multiple mitochondrial loci making it difficult to make direct comparisons unless secondary sequencing of the mitochondrial loci is performed (Hurst & Jiggins, 2005; van Dijk et al., 2014). The ability to create mitochondrial genome assemblies by mapping NGS data to a reference genome allows for contemporary high-resolution sequencing to continue without sacrificing the ability to compare to historic mitochondrial based work, which is of particular importance in a system as ecologically and economically important as *C. virginica*.

Here we demonstrate a method to: (1) assemble mitochondrial genomes with pooled restriction site associated DNA sequences (ezRAD; Toonen et al., 2013) from the northern Gulf of Mexico *C. virginica* population, (2) confirm that the mitochondrial genomes generated by pooled ezRAD are reliable by testing whether it conforms to expectations of mitochondrial DNA, and (3) assess the performance of population genetic inferences by comparison with a previously unpublished survey of genetic diversity with ND4 mtDNA using Sanger sequencing of individuals. Finally, we perform a population genetic analysis on the full mitochondrial genomes to evaluate genetic diversity and population structure across the mitochondrial genome of *C. virginica* within the northern Gulf of Mexico population, in the region where the population boundary is currently shifting north (King, Ward & Zimmerman, 1994; Varney et al., 2009; Anderson et al., 2014).

## Methods

### Collection and RADseq sample preparation

Fifty-eight *C. virginica* oysters were collected in the summer of 2013 from three bays along the mid-Texas Gulf coast: Copano Bay, San Antonio Bay, and Matagorda Bay (Fig. 1B). DNA from *C. virginica* mantle tissue was extracted using the Omega E-Z 96^®^ Tissue DNA Kit. Gel electrophoresis on a 1% agarose gel in 1x Tris-acetate-EDTA (TAE) was used to assess the length distribution of isolated DNA fragments in all samples. Beckman-Coulter SPRIselect paramagnetic beads were used to isolate high molecular weight DNA using 0.4x bead to sample reaction ratio. Inspection of size-selected samples was conducted using gel electrophoresis and samples exhibiting successful removal of low molecular weight DNA were used for ezRAD library preparation (Toonen et al., 2013). DNA concentrations were obtained with the Biotium AccuBlue High-Sensitivity fluorescence assay on a Molecular Devices SpectraMax M3 plate reader. Sample DNA concentrations were then equalized and pooled (9–10 individuals/pool, 2 pools/bay). The DNA was not barcoded by individual and thus we were unable to assign sequence reads to individuals, only to pools of individuals. Pooled ezRAD libraries were prepared with the Illumina TruSeq Nano DNA LT kit (Toonen et al., 2013) in the Genomics Core Laboratory at Texas A&M University - Corpus Christi. Modifications to the ezRAD protocol, such as incorporating the with-bead technique (Fisher et al., 2011), were used to reduce cost and minimize losses of DNA. Pools of genomic DNA were purified with Beckman-Coulter AmpureXP beads, eluted with nanopure water and digested with the isoschizomers, MboI and Sau3AI. AmpureXP beads remained in the samples and were reactivated with PEG (3M NaCl in 20% polyethylene glycol) upon completion of each reaction step. Samples were then processed following the Illumina TruSeq DNA Sample Prep Kit protocol at 1/3rd reaction volumes, and libraries were quantitated using the Kapa Library Quantification Kit on an Applied Biosystems StepOnePlus Real-Time PCR system. Library concentrations were normalized, and the libraries were sent to the Genomic Sequencing and Analysis Facility at the University of Texas at Austin for paired-end 100 bp sequencing on an Illumina HiSeq 2500.

### Sequence processing and mitochondrial genome assembly

After sequencing, fastqc (Andrews, 2010) was used to assess the quality of the sequences. Trim Galore! (Krueger, 2015) was used to remove adapters, restriction site sequences, base pairs with a Phred quality score below 15, and any reads that were below 50 base pairs in length after trimming. The trimmed reads were mapped to the existing Atlantic *C. virginica* mitochondrial genome using the bwa mem Burrows-Wheeler Aligner (Li & Durbin, 2010) to assemble pooled-genome-assembled genomes (PAGs) for each of the six pools of Texas oysters. All discussion of genomes from here forward refers to mitochondrial genomes. To differentiate between a genome assembly of a single individual and multiple individuals, we followed the terminology of Parks et al. (2017); metagenome-assembled genomes, MAGs) and termed genomes assembled from multiple individuals as pooled-genome-assembled genomes (PAGs). Prior to genotyping, alignments were filtered if they did not exceed an alignment score threshold of 50. Variants (SNPs) were called using freebayes with the ploidy-aware -j option (Garrison & Marth, 2012) and filtered if the overall depth of coverage was less than 10 or the PHRED quality was less than 30. Additionally, all indels and SNPs containing more than one alternative allele were filtered from this analysis. A northern Gulf of Mexico consensus PAG was constructed from the six pooled libraries using Cavener’s (1987) method. Bases with a read depth less than 3 were considered to not have been reliably sequenced and were excluded from creation of the consensus sequence. The northern Gulf of Mexico consensus PAG was annotated based on the existing Atlantic mitochondrial genome to investigate the relative number of SNPs in coding *versus* noncoding regions of the genome.

Pool-specific consensus PAGs were generated by assigning each position to the base representing more than 50% of the sample within the pool. Any base that was not sequenced in a pool was coded as N. If a position had exactly 50% of the reference and alternate base, then IUPAC nucleotide codes were used.

### Mitochondrial genome structure

We tested if the Gulf of Mexico consensus PAG met Chargaff’s second rule of parity (Chargaff, 1951; Rudner, Karkas & Chargaff, 1968) using a pair of *χ*^2^ tests to determine if the frequency of adenine is similar to thymine and if the frequency of guanine is similar to cytosine. To compare the percentage of adenine and thymine to the reference mitochondrial genome from the Atlantic, we performed an exact binomial test, testing if the observed AT frequency in our consensus sequence differed from that found in the reference genome.

As a method of confirming the validity of the PAGs, we tested the well-established hypotheses that there will be relatively more SNPs in non-coding regions of the genome and in the third-codon position of coding regions. To test if the PAGS follow these hypotheses, the frequency of SNPs was determined based on both codon position and sequence type (control, other non-coding, gene, tRNA, or rRNA regions of the sequence) and was analyzed using a *χ*^2^ test based on the null hypothesis that each position and sequence type is equally likely to contain a SNP based on the frequency of occurrence within the genome.

### Population genetics

We performed a basic population genetic analyses at the continental scale, comparing the level of genetic differentiation between the Atlantic and Gulf of Mexico and at the local scale investigating population structure among sampled Texas Bays near the population break between the Gulf of Mexico and Central/South American *C. virginica* populations. First, Kimura’s two-parameter genetic distance was calculated using the R package ape (Kimura, 1980; Paradis & Schliep, 2018) to measure the level of genetic differentiation between the Atlantic and Gulf of Mexico consensus sequences. We additionally, created a haplotype network of the six consensus PAGs and the reference Atlantic genome using only the positions with a single base present across all seven genomes using the popart implementation of the TCS network algorithm (Clement et al., 2002; Leigh & Bryant, 2015).

To analyze the population diversity and structure among the three bays we calculated the average nucleotide diversity across all bays (Nei & Li, 1979). We also performed an AMOVA to calculate the global *F*_CT_ among bays (Excoffier, Smouse & Quattro, 1992). Due to the pooled nature of the mitochondrial sequencing, we also calculated the *F*_SC_ of pools within bays. The AMOVA was performed using the pegas implementation within poppr (Paradis, 2010; Kamvar, Tabima & Grünwald, 2014).

A maximum-likelihood phylogenetic tree was created using one representative mitochondrial genome from each *Crassostrea* species available on NCBI, the consensus Gulf of Mexico PAG derived here, and *Ostrea* as an outgroup. The whole mitogenome markers were aligned and trimmed to a shared 24,715 bp core region using msa (Bodenhofer et al., 2015), which includes insertions. The best model of nucleotide evolution as identified by minimizing BIC and determined to be a GTR+G+I model. Initial trees were determined using neighbor joining with stochastic branch swapping and nearest neighbor interchange used to identify the maximum-likelihood tree. Branch support was assessed using 1,000 bootstrap replicates and stochastic branch rearrangement. Determination of the best model of nucleotide evolution and tree construction and bootstrapping was performed using phangorn (Schliep, 2011).

### Collection and sanger sequencing

To compare the diversity and population structure results from PAGS with those obtained with traditional sequencing, we utilized a previously unpublished study that targeted NADH dehydrogenase subunit 4 gene (ND4) using PCR and Sanger sequencing. This locus was originally selected due to its high degree of variability, but here it was a convenient dataset to compare results from the different methodologies. Oysters were purchased in 2007 from local suppliers which were harvested from the same three bays as the sampling for RAD sequencing (Copano, Matagorda, San Antonio Bays). Oysters were refrigerated or frozen prior to being shucked and stored in 70% ethanol. Total genomic DNA was extracted from approximately 25 mg of gill and/or mantle tissue from up to 26 oysters from each location (Copano: 23, Matagorda: 26, San Antonio: 21) using a Qiagen DNeasy Blood and Tissue Kit (Qiagen, Valencia, CA, USA). The ND4 was amplified using the following primers: F1: 5′-TCAGATTATTGCGATGACTAATGC-3′and R1: 5′- GTGGCCCACAAATCTCACTTT -3′and F2: 5′-TCAGATTATTGCGATGACTAATGC-3′and R2: 5′-GTGGCCCACAAATCTCACTTT-3′. Primers were amplified using the following conditions: 1X GoTaq Green (Promega), 1µM each primer, approximately 10 nM DNA in a 20 µL reaction. Thermocycling conditions consisted of an initial denaturation step of 2 min at 95 °C, 35 cycles of 95 °C for 30 s, 55 °C for 30 s, and 72 °C for 1 min, followed by a final elongation step at 72 °C and an indefinite hold at 4 °C. PCR product was then sent to MCLabs (San Francisco, CA USA) for purification and sequencing in both directions with the same primers. Sequences were cleaned and aligned in Sequencher Version 4.8 (Gene Codes, Ann Arbor, MI, USA) and trimmed to 1149 bp. All sanger-sequenced ND4 haplotypes can be found on GenBank (Accession: JN208241 –JN208352).

### Comparison of ND4 results between Sanger sequencing and RAD sequencing

To compare the results from the RAD and Sanger sequencing we repeated the calculations of nucleotide diversity and among bay population structure using both the ND4 locus extracted from the PAGs and the Sanger sequenced ND4 locus. We compared the resulting nucleotide diversities using 10,000 Monte Carlo simulations to determine if the observed difference in estimated nucleotide diversities is significant (North, Curtis & Sham, 2002). Due to differences in sampling design, we compared the *F*_ST_ derived from Sanger sequencing with the *F*_CT_ derived from the ND4 locus of the PAGs generated with RAD sequencing.

## Results

**Figure 2:**
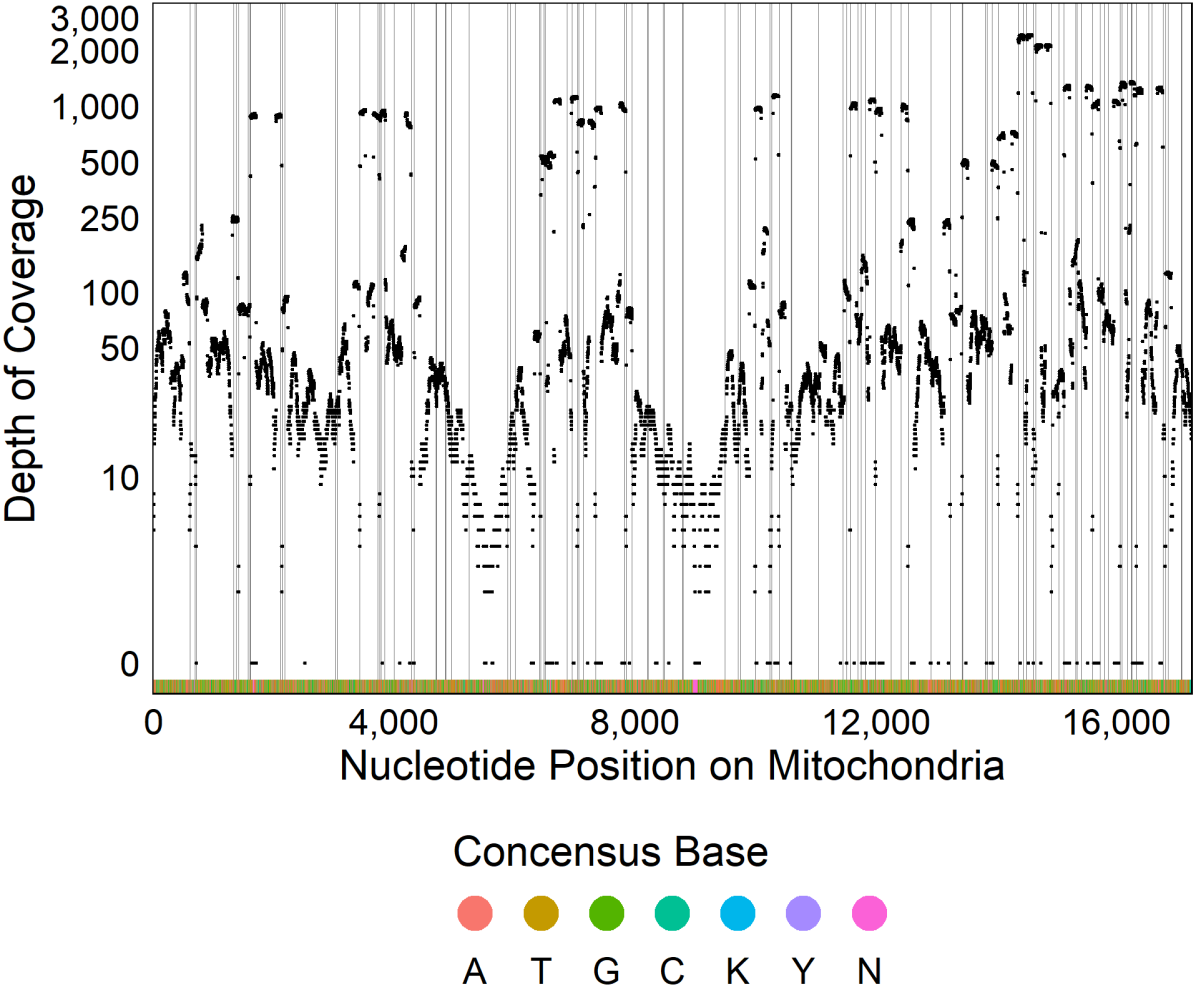
Sequencing coverage across mitochondrial genome. Plot of the depth of coverage (y) at each position in the mitochondrial genome (x). Vertical lines display restriction sites with the colors at the bottom of the figure showing the consensus DNA sequence.

The optimized ezRAD library preparation protocol resulted in 97% of the 38,180,502 sequence reads passing quality trimming. After sequence trimming, 95% of reads were between 91 and 94 base pairs in length. Of these reads, 47,213 (0.13%) mapped to the Delaware oyster mitochondrial genome from the Atlantic population, and the remaining reads were assumed to be from the *C. virginica* nuclear genome. We recovered 98% of the *C. virginica* mitochondrial genome, with only 308 out of 17,244 nucleotides sequenced in fewer than two reads (Fig. 2). After filtering, we detected 661 SNPs with a median depth of coverage of 69 and 16,275 monomorphic bases with a median depth of coverage of 45.

### Composition of the mitochondrial PAGs

The northern Gulf of Mexico *Crassostrea virginica* mitochondrial consensus PAG contains 17,244 base pairs, with a nucleotide composition of 4,473 adenine (26%), 5,854 thymine (34%), 2,782 cytosine, (16%), 3,824 guanine (22%), 308 *A*/*T*/*C*/*G* (1.8%), one A/G (0.01%), and two C/T (0.01%). The nucleotide composition did not follow Chargaff’s second rule of parity (A%∼T% *χ*^2^_(1)_ = 184.68, *p* <  < 0.001, G%∼C% *χ*^2^_(1)_ = 164.36, *p* <  < 0.001, (Chargaff, 1951; Rudner, Karkas & Chargaff, 1968) where purine - pyrimidine pairs are expected to occur in equal proportions (A%∼T% and G%∼C%), but violations of Chargaff’s rule are commonly observed in other mitochondrial genomes (Nikolaou & Almirantis, 2006; Mitchell & Bridge, 2006). Indeed, the Delaware mitochondrial genome from the Atlantic population had very similar nucleotide composition (A: 26%, T: 35%, C: 16%, G: 22%) and also did not follow Chargaff’s second rule of parity (A%∼T% *χ*^2^_(1)_ = 115.44, *p* <  < 0.001, G%∼C% *χ*^2^_(1)_ = 88.72, *p* <  < 0.001). There was an overabundance of thymine (33.95%) relative to adenine and an under-abundance of cytosine (16.13%) relative to guanine. The total number of AT base pairs was greater than that of GC base pairs, which was expected because it is known that the non-coding control region is AT-rich to facilitate strand separation at the onset of DNA replication. The AT composition for the northern Gulf of Mexico mitochondrial genome was 61.0% (95% CI 60.2%–61.7%) which is not significantly different from the Atlantic mitochondrial genome (61.1%, *p* = 0.765, Milbury & Gaffney, 2005).

**Figure 3:**
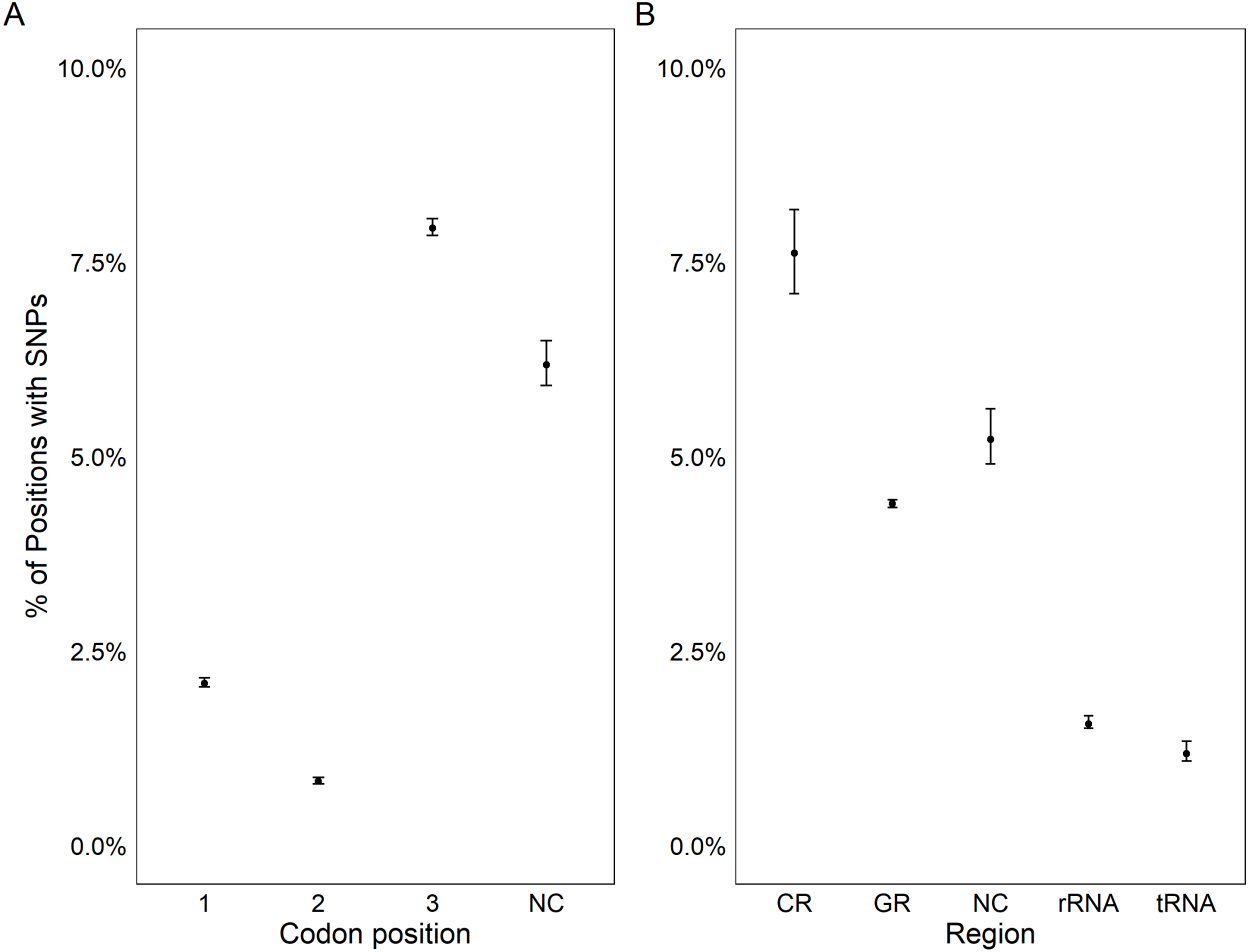
Genetic variation in genomic features. Plot showing the percentage positions containing SNPs found in each codon position (A) and region (B). Points represent the observed percentage of SNP loci with error bars showing 95% confidence intervals. CR stands for the control region, NC stands for non-coding region, and GR stands for gene region.

**Figure 4:**
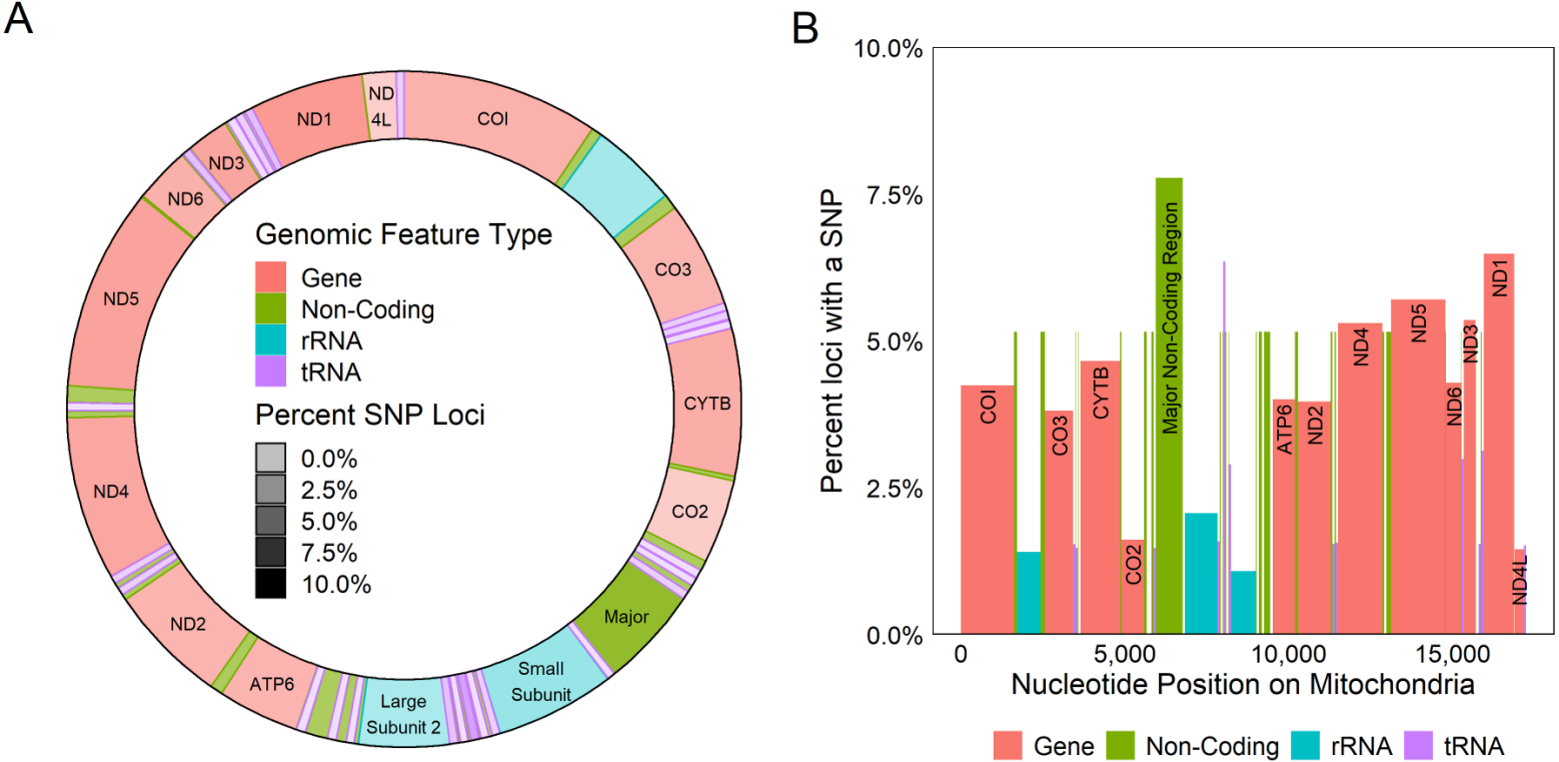
Genetic variation across mitochondrion. Diagram of the circular (A) and linear (B) mitochondrion indicating the locations of genomic features and the percent of the loci observed to contain a SNP within each gene. Color indicates the type of the genomic feature.

All coding regions of the mitochondrial genome had at least 97% sequencing coverage except the second location encoding tRNA-Met which was sequenced across 44.8% of the sequence (Table 1). Polymorphisms occurred within all protein and rRNA coding regions and 12 of the 23 tRNA coding regions. Polymorphisms occurred more frequently than expected in both non-coding regions (6.2%) and the third position of codons (7.9%) and less frequently in the first (2.1%) and second positions (1.0%, *χ*^2^_(6)_ = 632.11, *p* < 0.0001, Fig. 3A). Additionally, among non-coding regions the control region had the highest proportion of SNPs (7.6%) while all other non-coding regions had a 5.2% frequency of SNPs. Both non-coding regions had higher genetic diversity than coding regions. Further we found that protein coding genes had a higher frequency (4.4%) of SNPs than either tRNA (1.2%) or rRNA (1.6%) encoding regions which were similar (*χ*^2^_(6)_ = 331.19, *p* < 0.0001, Figs. 3B, 4).

**Table 1:** Median coverage and number of each type of base found in each genetic feature of the mitochondrial genome.

Feature type	Feature	Median coverage	Number of invariant bases	Number of SNPs	Number of unsequenced bases	Percentage of feature sequenced	SNP percent
Non-Coding	Control Region	36	728	60	44	94.7	7.6
Non-Coding	Other Non-Coding	21	1126	62	77	93.9	5.2
Gene	ATP6	40	647	26	2	99.7	3.9
Gene	COX2	10	662	11	20	97.1	1.6
Gene	COX3	25	840	32	0	100.0	3.7
Gene	COXI	52	1547	66	10	99.4	4.1
Gene	CYTB	49	1145	54	14	98.8	4.5
Gene	ND1	212	855	57	24	97.4	6.2
Gene	ND2	30	945	38	13	98.7	3.9
Gene	ND3	992	333	18	3	99.2	5.1
Gene	ND4	68	1256	68	26	98.1	5.1
Gene	ND4L	33	276	4	0	100.0	1.4
Gene	ND5	80	1569	90	9	99.5	5.4
Gene	ND6	45.5	439	19	4	99.1	4.1
rRNA	Large Subunit 1	40	711	10	0	100.0	1.4
rRNA	Large Subunit 2	10	730	8	10	98.7	1.1
rRNA	Small Subunit	90.5	956	20	13	98.7	2.0
tRNA	tRNA-Ala	63	64	2	0	100.0	3.0
tRNA	tRNA-Arg	43	65	1	0	100.0	1.5
tRNA	tRNA-Asn	38	70	0	0	100.0	0.0
tRNA	tRNA-Asp	23	69	2	0	100.0	2.8
tRNA	tRNA-Cys	26	63	4	0	100.0	6.0
tRNA	tRNA-Gln	151	65	2	2	97.1	3.0
tRNA	tRNA-Glu	101	69	0	0	100.0	0.0
tRNA	tRNA-Gly	47	65	0	0	100.0	0.0
tRNA	tRNA-His	101	64	1	0	100.0	1.5
tRNA	tRNA-Ile	926	65	1	0	100.0	1.5
tRNA	tRNA-Leu 1	8	67	0	0	100.0	0.0
tRNA	tRNA-Leu 2	96	71	0	0	100.0	0.0
tRNA	tRNA-Lys	14	68	0	0	100.0	0.0
tRNA	tRNA-Met 1	80	63	1	0	100.0	1.6
tRNA	tRNA-Met 2	7	30	0	37	44.8	0.0
tRNA	tRNA-Phe	70	65	1	0	100.0	1.5
tRNA	tRNA-Pro	13	68	1	0	100.0	1.4
tRNA	tRNA-Ser 1	5	71	0	0	100.0	0.0
tRNA	tRNA-Ser 2	3	74	0	0	100.0	0.0
tRNA	tRNA-Thr	57	68	1	0	100.0	1.4
tRNA	tRNA-Trp	27	66	1	0	100.0	1.5
tRNA	tRNA-Tyr	17	75	0	0	100.0	0.0
tRNA	tRNA-Val	15	65	0	0	100.0	0.0

### Phylogeny, haplotype network & population structure

**Figure 5:**
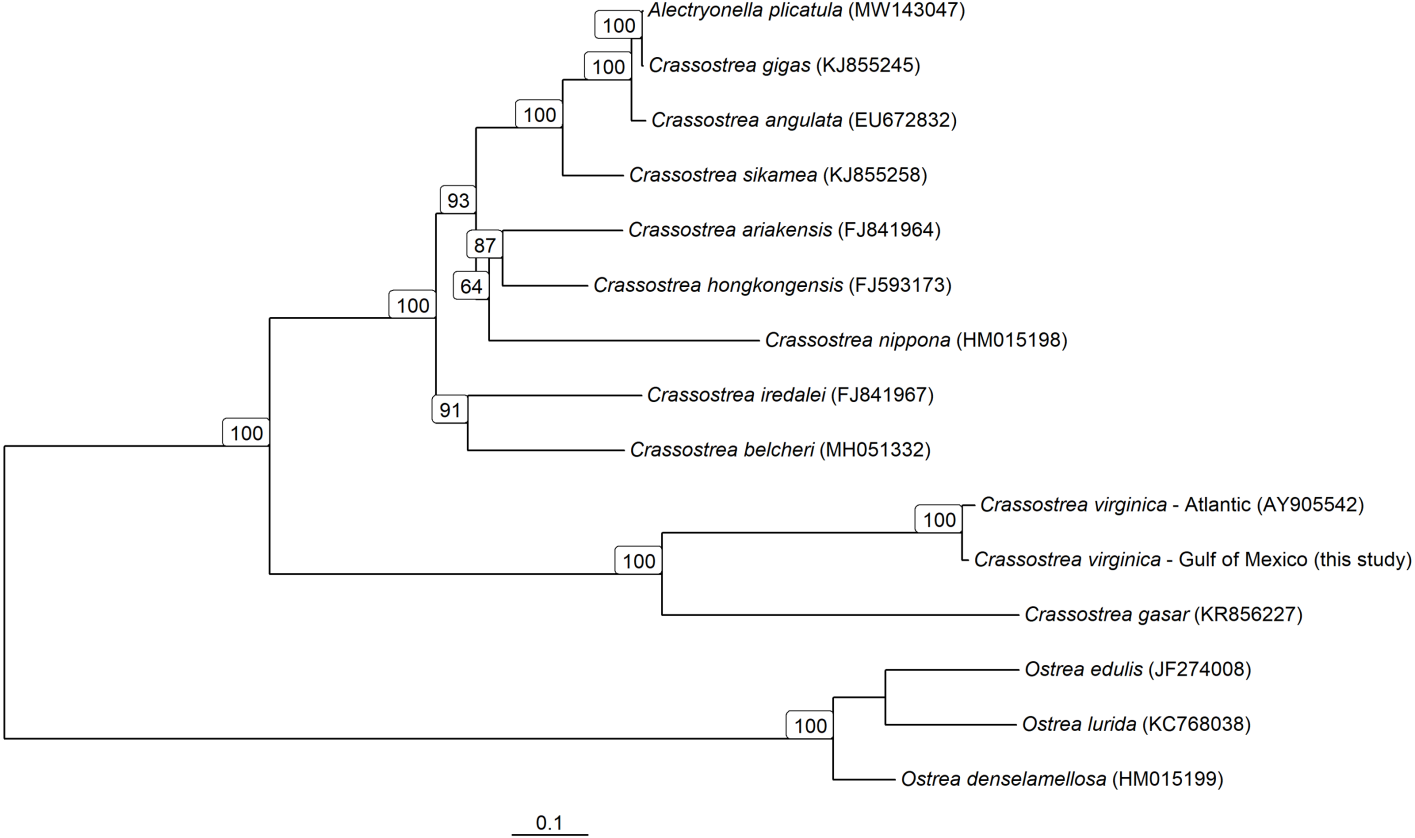
Mitochondrial Genome based phylogeny of *Crassostrea spp*. Maximum-likelihood phylogenetic relationships among *Crassostrea spp.* based on their mitochondrial genomes. Text in parentheses after species names are the accession numbers for each sequence used and numbers on the tree represent bootstrap support values (any bootstrap support values less than 60% are not displayed). The scale bar represents the proportion of sites that segregate the partial genomes.

**Figure 6:**
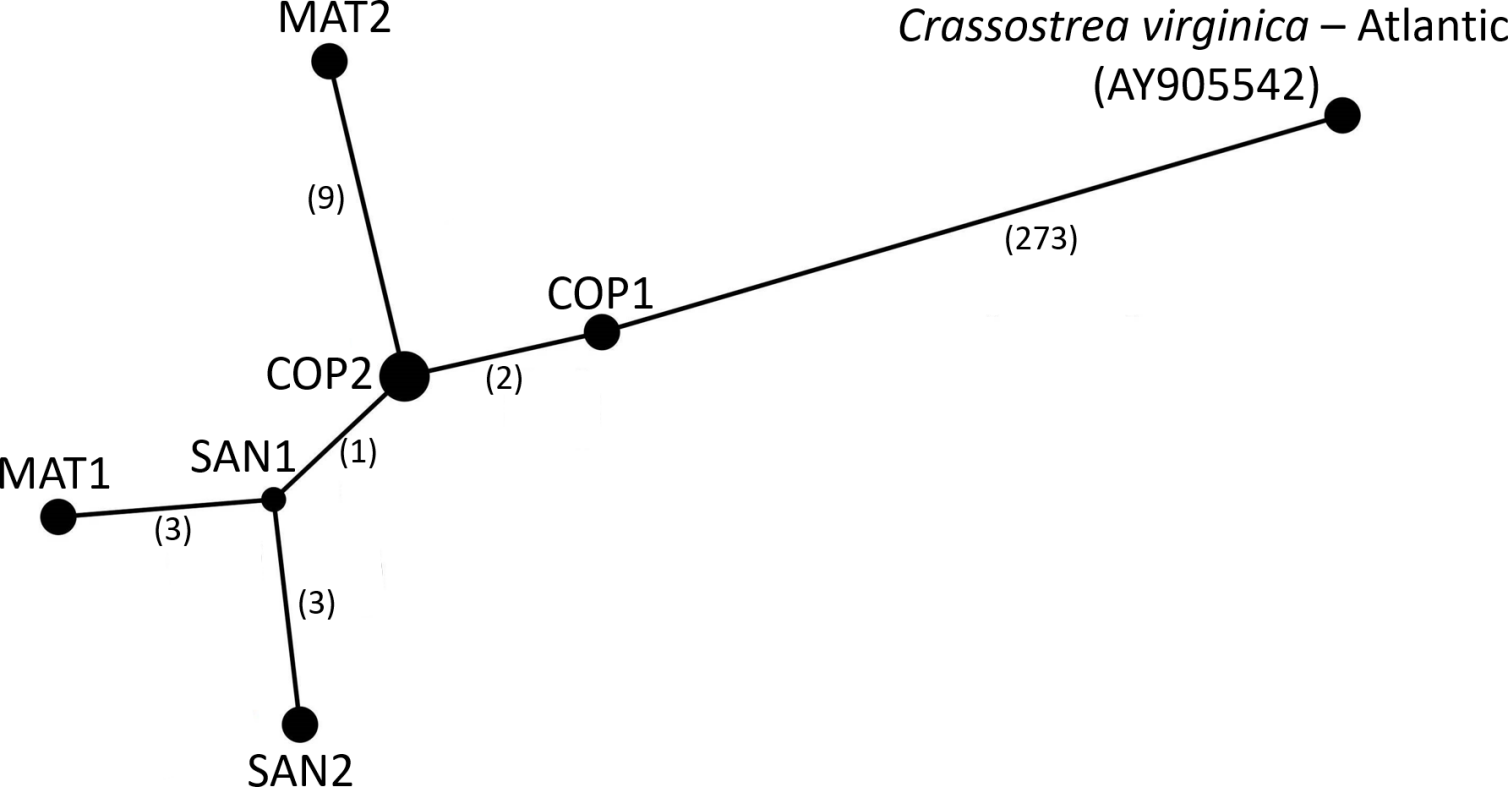
Haplotype Network of *Crassostrea virginica* in the Gulf of Mexico and Atlantic. Haplotype network of the sampled pooled mitochondrial PAGs compared with the reference genome. Numbers in parentheses indicate the number of base step mutations distinguishing the haplotypes.

The consensus PAG from the northern Gulf of Mexico was found to be sister to the *C. virginica* mitochondrial genome representing the northern Atlantic population (Fig. 5) with a genetic distance of 0.022 and high bootstrap support (100%). In agreement with other studies, we found the most closely related species to *C. virginica* is *C. gasar* (Fig. 5, Cavaleiro et al., 2016; Wang et al., 2021; Salvi et al., 2021). The *Alectryonella plicatula* positioned sister to *Crassostrea gigas* is a possibly misidentified *C. gigas* specimen (Salvi et al., 2021). The clade containing *C. gigas*, *C. angulata*, *C. sikamea*, *C. ariakensis*, *C. hongkongensis*, *C. nippona*, *C. iredalei*, and *C. belcheri* has been alternatively reclassified as the genus *Magallana* (Salvi, Macali & Mariottini, 2014; Salvi & Mariottini, 2016). The six consensus PAGs from the northern Gulf of Mexico were much more closely related to each other (1–14 mutational differences; Fig. 6), than to the Atlantic mitochondrial genome (273–284 mutational differences). Using the six consensus bay PAGs we observed a nucleotide diversity of 0.00241 (±0.0012) and found no population structure among bays (*F*_CT_ = −0.037; *p* = 0.999) but did observe a significant pool effect within bays (*F*_SC_ = 0.198; *p* < 0.001), as was the case for ND4.

### Comparison of genetic diversity and structure between PAGs and sanger sequence (ND4)

There was no evidence of a significant difference in the nucleotide diversity calculated from samples using Sanger sequencing or the ND4 locus extracted from the PAGs ($\hat {\pi }$ = 0.00427 ± 0.0023, $\hat {\pi }$ = 0.00302 ± 0.0017 respectively, *p* = 0.479). Neither exhibited statistically significant population structure among bays (Sanger: *F*_ST_ = 0.0059, *p* = 0.191; PAGs: *F*_CT_ = −0.0130; *p* = 0.725). However, we did find a significant pool effect within bays (*F*_SC_ = 0.107; *p* = 0.002) when analyzing only the ND4 locus.

## Discussion

### Assessment of mitochondrial PAG validity

In comparison to the first, unoptimized, ezRAD datasets (Toonen et al., 2013), the optimized library preparation protocol employed here yields higher quality data (2.78% of reads were filtered for low-quality *versus* 46.00 ± 7.21% SE filtered for low-quality by Toonen et al., 2013). The increased quality of the resulting data was suitable to assemble mitochondrial genome sequences as a byproduct. The ability to assemble mitochondrial genomes from ezRAD data has also been demonstrated for corals (Tisthammer et al., 2016). The small percentage of reads that mapped to the *C. virginica* mitochondrial genome (0.13%) was expected because mitochondrial DNA comprised a small percentage of the total targeted RAD loci. Indeed, the nuclear genome of *C. virginica* has approximately 675 million nucleotides (Goldberg et al., 1975) and the mitochondrial genome is only 17,244 nucleotides (Milbury & Gaffney, 2005).

The assembly of the pooled mitochondrial genomes from several oysters produced PAGs with patterns of genetic variation that met *a priori* expectations. In comparison to the previously published mitochondrial genome, there was a nearly identical distribution of nucleotides (61.0% A/T compared to 61.1% A/T) and nucleotide diversity. While Chargaff’s (1968) rule of parity was violated, it is also violated by the published *C. virginica* genome (Milbury & Gaffney, 2005). This result is ubiquitous in animal mitochondria and has been linked to the method of mitochondrial replication in animals (Nikolaou & Almirantis, 2006). To further validate the PAGs, we tested the hypotheses that there should be increased genetic diversity in non-coding regions (Kimura, 1991) and in the third codon position (wobble position; Crick, 1966; Kimura, 1991). The PAGs met the predictions of increased genetic diversity in both non-coding regions and in the third codon position within coding regions (Fig. 3).

There was no evidence for rearrangements of the mitochondrial genome of *C. virginica* in the northern Gulf of Mexico population relative to that in the North Atlantic, nor were they expected. Ren et al. (2010) found that six Pacific species of *Crassostrea* exhibited no gene rearrangements, and we know of no examples of rearrangements within a *Crassostrea* species. Ren et al. (2010) did identify 6 transpositions and 3 duplications of tRNAs between Pacific *Crassostrea* spp. and *C. virginica*, demonstrating that there can be differences among species within the genus. In the present study, there was a low percent of tRNA-Met 2 sequences with ≥3x coverage (48%) relative to other regions (93.9–100%, Table 1), but visual inspection of the alignment maps revealed that there were a small number of well aligned reads across pools that anchor tRNA-Met 2 in the same position as in the North Atlantic *C. virginica*. The only other locations that were not spanned by any reads had the recognition site of the restriction enzymes we used to digest the genome, and thus we would not expect reads to span those positions unless they had polymorphisms that affected their digestion. It should be noted that constructing PAGs by mapping to a reference genome will generally only work well when there are no chromosomal rearrangements, otherwise *de novo* assembly would be required.

### Comparison of RAD PAGs to sanger amplicons and genome assembly

Both the population genetic structure inferred with Sanger sequenced ND4 amplicons and the evolutionary reconstructions based on whole mitogenomes produced the similar results when using RAD PAGs or the original data source. In the evolutionary reconstruction of *Crassostrea* mitochondrial genomes, the Gulf of Mexico consensus PAG and northern Atlantic genome were statistically significant sister haplotypes, demonstrating the utility of PAG data for phylogenetic analysis. Further, there were no significant differences between the estimated nucleotide diversity or population structure in the ND4 locus using either traditional Sanger sequencing methods or the mitochondrial PAGs derived from RAD sequencing data. It is important, however, to process multiple pools from the same location to flag artifacts associated with pooled RAD sequencing. Indeed, there was structure detected between two pools of RAD data from the same bay likely due to artifacts, but no structure was detected between bays, as with the Sanger data. The consistency of these suggests that future research using NGS techniques like ezRAD that allow mitochondrial reconstruction can be directly compared to historic research using only mitochondrial loci. This is likely to be particularly helpful in documenting temporal shifts in spatial population structure or genetic diversity as it allows for comparisons to previously published research. It is also useful for identifying the species given that the present DNA barcoding databases are mostly comprised of mitochondrial sequences for marine animals, and it can be problematic to genetically assign species identity with RAD data sets from non-model species.

While it would have been ideal to directly Sanger sequence the same individuals that were subjected to ezRAD, we did not have the resources to do this. However, demonstrating a similar lack of population genetic structure across the same bays, albeit at different time points, and comparing phylogenetic divergence within the northern Gulf of Mexico PAGs with their divergence from the northern Atlantic mitochondrial genome constructed from Sanger data does provide support that the PAGs are valid.

### Phylogeny and population genetic structure of *C. virginica*

The northern Gulf of Mexico *C. virginica* PAGs were significantly differentiated from the northern Atlantic *C. virginica* genome. Among the Texas PAGs, we found an average genetic divergence of 0.049%, which was 44-fold less than between Texas and the northern Atlantic, suggesting the possibility of cryptic speciation and warranting further investigation (Hebert et al., 2004; Ward, 2009).

When using the complete PAGs, there was no population structure among the surveyed Copano, Matagorda, and San Antonio bays. South Texas bays and estuaries are typified by long residence times due to minimal freshwater inflow and tidal mixing with the bays we surveyed ranging from ∼50 days up to more than 350 days (Solis & Powell, 1998). Given that the larval duration of *C. virginica* is estimated to be only 15 –25 days, our initial assumption would be minimal gene flow between distinct populations in the bays (Kennedy, 1996). However, there is primarily wind driven direct exchange of surface water between bays through the Gulf Intracoastal Waterway (Schroeder & Wiseman, 1998; East, 2001) which is likely to promote geneflow. This may be the mechanism facilitating transport of larvae near the surface between bays without entering the Gulf of Mexico. The distribution of *C. virginica* larvae in the water column is a result of the specific physical characteristics of the system (*e.g.*, stratification, flow velocities) and their interaction with biological aspects (*e.g.*, swimming, selective feeding, larval stage; (Dekshenieks et al., 1996; Finelli & Wethey, 2003; Kim et al., 2010). Hence, there might be occasions which facilitate the exchange of larvae between bays directly through the linked surface waters.

## Conclusion

As reduced representation genome sequencing becomes more commonly used in population genetic research, it becomes difficult to incorporate previous findings using lower density markers. Various mitochondrial markers have seen widespread use in population and phylogenetic studies as well as species delimitation and other research avenues. Here we demonstrated the validity of mitochondrial sequence data extracted from ezRAD libraries for both population and phylogenetic inquiry. This allows studies using the higher density markers the ability to compare results to previous research using mitochondrial markers.

## Supplemental Information

10.7717/peerj.12205/supp-1 Supplemental Information 1 All non-sequence data and code for analysis Collection and pooling data is found in ”oyster_pooling.csv”. The unfiltered vcf file analyzed are found in ”oyster_unfiltered.vcf”. Sanger sequencing collection and sequence data are found in ”Oyster ND4 Haplotypes by location.xlsx”. Scripts to map ezRAD reads to the reference genome, filter poorly aligned reads, and perform vcf filtering and full analysis are found in bash and R scripts.

## Additional Information and Declarations

### DNA Deposition

The following information was supplied regarding the deposition of DNA sequences: The raw ezRAD sequences are available at GenBank: SAMN18515112–SAMN18515117. The Sanger sequenced NADH dehydrogenase subunit 4 gene are available at GenBank: JN208241 –JN208352.

### Data Availability

The following information was supplied regarding data availability: All data and code are available in the Supplemental File.
